# Influence of nanoparticles aggregation and Lorentz force on the dynamics of water-titanium dioxide nanoparticles on a rotating surface using finite element simulation

**DOI:** 10.1038/s41598-023-31771-w

**Published:** 2023-03-22

**Authors:** Bagh Ali, Imran Siddique, Hijaz Ahmad, Sameh Askar

**Affiliations:** 1 School of Mechanical Engineering and Automation, Harbin Institute of Technology, Shenzhen, 518055 China; 2 Faculty of Computer Science and Information Technology, Superior University, Lahore, 54000 Pakistan; 3 Department of Mathematics, University of Management and Technology, Lahore, 54770 Pakistan; 4 Section of Mathematics, International Telematic University Uninettuno, Corso Vittorio Emanuele II, 39, 00186 Rome, Italy; 5 Department of Statistics and Operations Research, College of Science, King Saud University, P.O. Box 2455, Riyadh, 11451 Saudi Arabia; 6 Near East University, Operational Research Center in Healthcare, Near East Boulevard, 10, 99138 Nicosia/Mersin, Turkey

**Keywords:** Engineering, Mathematics and computing, Nanoscience and technology

## Abstract

This communication briefings the roles of Lorentz force and nanoparticles aggregation on the characteristics of water subject to Titanium dioxide rotating nanofluid flow toward a stretched surface. Due to upgrade the thermal transportation, the nanoparticles are incorporated, which are play significance role in modern technology, electronics, and heat exchangers. The primary objective of this communication is to observe the significance of nanoparticles aggregation to enhance the host fluid thermal conductivity. In order to model our work and investigate how aggregation characteristics affect the system’s thermal conductivity, aggregation kinetics at the molecular level has been mathematically introduced. A dimensionless system of partial-differential equations is produced when the similarity transform is applied to a elaborated mathematical formulation. Thereafter, the numerical solution is obtained through a well-known computational finite element scheme via MATLAB environment. When the formulation of nanoparticle aggregation is taken into consideration, it is evident that although the magnitude of axial and transverse velocities is lower, the temperature distribution is enhanced by aggregation.

## Introduction

Nanofluids are fluids that are created by the homogenous dispersion of tiny particles of metal or metal oxides at the nanoscale^1^. The host fluid which contains the nanomaterials has a valuable influence on the dynamics of host fluid according to the experimental and numerical investigation^2,3^. The modern era researchers are fascinating due to wide range of nanofluids application in every field of engineering and science^4^. The host fluid temperature enhanced due to mixture nano-sized particles inside the fluid and it is happen because of effective thermal transport of tiny particles which are responsible for enhancing heat transfer rate^5^. The tiny solid particles different shapes influence the host fluid dynamics and significance influence on host fluid temperature^6^. The different particles features inside the nanofluid flows have been investigated numerically, analytically, and experimentally by the research community^7^. Recent, several researchers investigated the different nature of nanoparticles to improve the host fluid thermal enhancement subject to different types of techniques and geometeries^8–10^. The inclusion of nano-particles in the base fluid resulted in a rapid rise in temperature, whereas the mono fluid had a smaller impact on temperature than the hybrid nanofluids^11^. The combination of base fluids and small solid fragments can improve the thermal properties of several fluids^12,13^.

In our daily life, the titanium dioxide is key component which is studied in metal oxide surface science as a crystalline oxides because of its capacities as a photocatalyst with sensibly high proficiency for water breakdown^14^. Titanium dioxide powders have assortments of applications, attributable to its ability to bestow whiteness furthermore, darkness to different things, including paints, papers,and beauty care products. The titanium dioxide has acquired incredible consideration as a promising photocatalyst because of its useful properties among the other photocatalysts, high compound strength, and minimal expense^15,16^. The numerous researchers presented titanium dioxide tiny particles used for the treatment of dangerous cancers types^17^ and many other aspect of human daily life^18^. Fujishima et al.^19^ specifically provided additional details regarding the outstanding titanium dioxide photocatalyst material for environmental purification.

The aggregation of nanoparticles have a significant impact on both thermal and rheological properties. In order to construct a passageway that has a lower thermal resistance, the aggregated particles typically form linear chains^20^. Because of this, heat can be moved very quickly through the nanoparticles clusters. In addition to the greater influence volume of aggregations compared to that of nanoparticles, can increase the nanofluid’s heat conductivity^21^. Experiments have shown that particle aggregation can increase the efficient heat conductivity of nanofluids^22–24^. The nano-sized nanoparticles clustering have a remarkable influence to upgrade host fluid thermal properties and aggregated nanoparticles are more efficient heat transfer rate as compared to non-aggregated nanoparticles^25^. In light of practical applications like automobile radiators, printing inks, and polyester synthesis, as well as the significance of particles aggregation for enhancing heat transport, nanoparticles aggregation gain mush popularity now a days^26^. The aggregation inside the host fluid is influenced by both initial concentration and cluster size^27^. After that, the aggregated particles volume, number of nanoparticles per aggregated cluster, and the fractal dimensions of the size of the clusters were reported in references^28,29^. The significant applications of magnetohydrodynamic (MHD) engagement can be found in aerospace engineering, astrophysics, and medicine. Due to the connection between the electrically charged liquid and the magnetic field, numerous machines with MHD generators, boundary layer flow, bearings, and turbulent pumps are pretentious. Zeeshan et al.^30^ examined the significance of magnetic field subject to water contain titanium dioxide nanofluid and they found that the magnetic field has significance impact on the dynamics of nanofluid. The host fluid temperature enhanced due to higher strength of magnetic field and recede the fluid velocity^31^.

A review of the literature reveals that rotating nanofluid has received less attention subject to nanoparticle aggregation. As a result, the current manuscript concentrated on the transport phenomenon of Titanium dioxide nanoparticle aggregates in water-based fluids under the influence of an externally imposed magnetic field. Through aggregation, the thermal conductivity of nanoparticles can be significantly improved. Additionally, nanoparticles come with a number of difficulties. Trying to figure out how a system’s molecular interaction, like aggregation kinetics, affects the fluid’s thermophysical properties is one of engineering’s biggest challenges. Through this literature, we learn how aggregation kinetics can affect heat transfer, generated temperature, and thermal conductivity. In light of previous research, we therefore investigated the effect of aggregation kinetics on a rotating nanofluid subject to lorentz force. The prime objective of this communication is to observe the role of nanoparticles aggregation to enhance the host fluid thermal conductivity. The FEM, a well-known computational scheme^32,33^, is used to solve MATLAB environment. When the influence of nanoparticle aggregation is taken into consideration, it is evident that although the velocity is lower, the temperature function is upgraded by aggregation. Thus, demonstrating aggregates’ significance and impact, as well as their value as a theoretical tool for future industrial and technological applications. The various types of boundary layer flow subject to different fluid related problems have been solved via finite element approach studied by^34^. This approach solved fluid problems rapidly, rapidly, and precisely. This report give the answers of below research questions:

1. What is influence of magnetic parameter on the characteristics of water-titanium dioxide nanofluid subject to titanium dioxide particles aggregation and non-aggregation?
2. What is impact of Coriolis force on the dynamic of water-titanium dioxide nanofluid subject to titanium dioxide particles aggregation and non-aggregation?
3. What impact do the magnetic and rotating parameters have on Nusselt number and skin friction factors in the presence of titanium dioxide particles aggregation and non-aggregation?

## Mathematical formulation

**Figure 1:**
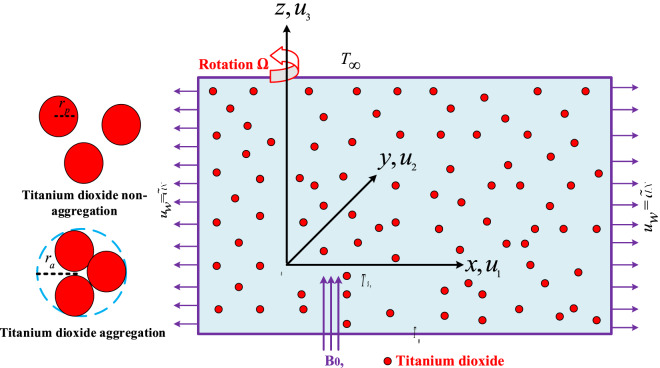
Flow configuration.

Take into consideration time dependent three-dimensional rotating water-based nanofluid that flows across a stretching subject to magnetic field. On an elongated surface, a three-dimensional, non-transient flow of magnetized nano liquid (TiO2) is considered, as depicted in Fig. 1. By taking into account the corresponding dynamic viscosity and thermal conductivity, the influence of nanoparticle aggregation is evaluated. The movement of nanoparticles suspended in liquid would result in aggregations that significantly impact the base fluids’ physical properties. The elaborated problem system is rotating in *z* direction, which is oriented transverse to xy plane, at an angular velocity of $\Omega$. The fixed origin *O*(*x*, *y*, *z*) has been chosen, with the x-axis representing the movement of the stretching surface, the y-axis representing the normal of the surface, and the z-axis representing transverse to the *xy* plane. In the axial (z-direction) direction, a static and uniform magnetic $B_0$ field is applied^35^. The ambient temperature is represented by $T_\infty$, and the surface temperature is represented by $T_w$. The components $u_1$, $u_2$, and $u_2$ show the velocity components along *x*, *y*, and *z* directions, respectively. Tables 1 and 2 details the aggregation and non-aggregation related physical properties of based fluid and nanoparticles. The governing equations of continuity, momentum, and temperature are given below^36,37^:

1 $$\begin{aligned}{} & {} {u}_{1x}+{u}_{2y}+{u}_{3z}=0, \end{aligned}$$

2 $$\begin{aligned}{} & {} \rho _{nf}({u}_{1t}+ {u}_1{u}_{1x} + {u}_2{u}_{1y} + {u}_3{u}_{1z} + 2\Omega {u}_2)= \mu _{nf}{u}_{1zz} - \sigma _{n_f}B_0^2u_1, \end{aligned}$$

3 $$\begin{aligned}{} & {} \rho _{nf}({u}_{2t}+ {u}_1{u}_{2x} + {u}_2{u}_{2y} + {u}_3{u}_{2z} - 2\Omega {u}_1)= \mu _{nf}{u}_{2zz}- \sigma _{n_f}B_0^2u_2, \end{aligned}$$

4 $$\begin{aligned}{} & {} {T}_t + {u}_1{T}_x + {u}_2{T}_y +{u}_3{T}_z = \alpha _{n_f}{T}_{zz}, \end{aligned}$$

where $\mu _{n_f},\ \rho _{nf},\ \alpha _{n_f}$, are the dynamic viscosity, fluid density, and thermal diffusivity, *T* represents the fluid temperature. The formulated problem boundary conditions are^38,39^:

5 $$\begin{aligned}{} & {} t <0: {u}_1 = 0,\ {u}_3 = 0,\ {u}_2 = 0,\ {T} = {T}_\infty , \end{aligned}$$

6 $$\begin{aligned}{} & {} t\ge 0: {u}_1 =\tilde{a}x,\ {u}_3= 0,\ {u}_2 = 0,\ {T} = {T}_s,\ as\ z=0, \end{aligned}$$

7 $$\begin{aligned}{} & {} t\ge 0: {u}_1\rightarrow 0,\ {u}_2\rightarrow 0, \ {T}\rightarrow {T}_\infty . \end{aligned}$$

Similarity transformations (see^36,38^):

8 $$\begin{aligned} \left. \begin{array}{ll} u_1= \tilde{a}x\frac{\partial F_1(\Gamma ,\eta )}{\partial \eta },\ u_2 = \tilde{a}xF_2(\Gamma ,\eta ),\ u_3 = -\sqrt{\tilde{a}\nu \Gamma }F_1(\Gamma ,\eta ),\\ \Gamma = 1-e^{-\zeta }, \eta = \sqrt{\frac{\tilde{a}xz^2}{\Gamma \nu }},\ \zeta = \tilde{a}t,\ T = \Theta (\Gamma ,\eta )(T_s-T_\infty )+T_\infty . \end{array}\right\} \end{aligned}$$

**Table 1:** Thermophysical properties model of titanium dioxide and water nanofluid^40^.

Properties	$$\rho$$ (kg/$$m^{3})$$	$$C_p$$ (J/kg K)	$$\kappa$$ (W/m K)
${\hbox {H}_2}\hbox {O}$	0991.1	4179.0	00.613
${\hbox {TiO}_2}$	4250.0	686.20	8.9538

**Table 2:** Thermophysical properties model of nanofluid^40,41^.

Properties	Aggregation case	Non-aggregation case
Viscosity $(\mu )$	$\frac{\mu _{n_f}}{\mu _{f}}={(1-\frac{\Phi _{ag}}{\Phi _{m}})^{2.5\Phi _m}}$	$\frac{\mu _{n_f}}{\mu _{f}}=\frac{1}{(1-\Phi )^{2.5}}$
Density $(\rho )$	$\rho _{n_f}=\rho _f(1-\Phi _{ag})+\Phi _{ag}\rho _s$	$\rho _{n_f}=\rho _f(1-\Phi )+\Phi \rho _s$
Heat capacity$(\rho C_p)$	$(\rho C_p)_{nf}=(\rho C_p)_f(1-\Phi _{ag})+\Phi _{ag}\frac{(\rho C_p)_s}{(\rho C_p)_f}$	$(\rho C_p)_{nf}=(\rho C_p)_f(1-\Phi )+\Phi \frac{(\rho C_p)_s}{(\rho C_p)_f}$
Thermal conductivity ($\kappa$)	$\frac{k_{n_f}}{k_f}=\frac{k_{ag}+2k_f-2\Phi _{ag}(k_f-k_{ag})}{k_{ag}+2k_f + \Phi _{ag}(k_f-k_{ag})}$	$\frac{k_{n_f}}{k_f}=\frac{k_s+2k_f-2\Phi (k_f-k_s)}{k_s+2k_f + \Phi (k_f-k_s)}$

In above table, $\Phi$ is nanoparticles volume fraction. In view of Eq. (8), Eq. (1) is satisfied and Eqs. (2–7) becomes non-linear PDEs into transformed coordinate systems ($\Gamma ,\eta$).

9 $$\begin{aligned} \frac{1}{\chi _1\chi _2}F_1'''+ 0.5\eta {F_1''}-0.5\Gamma \eta {F_1''}+\Gamma (F_1F_1''-F_1'^{2} - \frac{M^2}{\chi _2}F_1'+ 2\lambda {F_2}) -\Gamma (1-\Gamma )\frac{\partial {F_1}'}{\partial \Gamma }=0, \end{aligned}$$

10 $$\begin{aligned} \frac{1}{\chi _1\chi _2}F_2''+ 0.5\eta {F_2'}-0.5\Gamma \eta {F_2'}+\Gamma (F_1F_2'- 2\lambda {F_1}'- \frac{M^2}{\chi _2}F_2-F_1'F_2)-\Gamma (1-\Gamma )\frac{\partial {F_2}}{\partial \Gamma }=0, \end{aligned}$$

11 $$\begin{aligned} \frac{\chi _3}{\chi _4}\Theta '' + 0.5\eta (1-\Gamma )P_r\Theta ' +\Gamma {P_r}F_1\Theta '- \Gamma (1-\Gamma )P_r\frac{\partial \Theta }{\partial \Gamma }=0, \end{aligned}$$

12 $$\begin{aligned} \left. \begin{array}{ll} F_1(\Gamma ,\eta =0) = 0,\ F_1'(\Gamma ,\eta =0) =1, \ F_2(\Gamma ,\eta =0) = 0,\ \Theta (\Gamma ,\eta =0) = 1,\ \Gamma \ge = 0,\ at\ \eta =0,\\ F_1'(\Gamma ,\eta \rightarrow \infty )\rightarrow 0,\ F_2(\Gamma ,\eta \rightarrow \infty )\rightarrow 0,\ \Theta (\Gamma ,\eta \rightarrow \infty )\rightarrow 0,\ \Gamma \ge 0,\ as\ \eta \rightarrow \infty . \end{array}\right\} \end{aligned}$$

where

$$\begin{aligned} \chi _1&= {\left( 1-\frac{\Phi _{ag}}{\Phi _{m}}\right) ^{-2.5\Phi _m}},\ \chi _3 = \frac{k_{ag}+2k_f-2\Phi _{ag}(k_f-k_{ag})}{k_{ag}+2k_f + \Phi _{ag}(k_f-k_{ag})},\ \chi _2 = (1-\Phi _{ag})+\Phi _{ag}\frac{\rho _{ag}}{\rho _f},\\ \chi _4&= (1-\Phi _{ag})+\Phi _{ag}\frac{(\rho {C_p})_{ag}}{(\rho {C_p})_f}, \end{aligned}$$

and $\lambda = \frac{\Omega }{{a}}$ signifies rotating parameter, $M = \sqrt{\frac{\sigma _{n_f}B_o^2}{\rho _f\tilde{a}}}$ magnetic parameter, and $P_r = \frac{\nu }{\alpha _{n_f}}$ symbolize the Prandtl number. The physical parameters of Nusselt number, and skin friction coefficients are defined as:

13 $$\begin{aligned}{} & {} Nu = \frac{xq_w}{\kappa ({T}_s-{T}_\infty )}, \end{aligned}$$

14 $$\begin{aligned}{} & {} C_{f_x} = \frac{\tau _w^x}{\rho {u}_1^2}, \end{aligned}$$

15 $$\begin{aligned}{} & {} C_{f_y} = \frac{\tau _w^y}{\rho {u}_1^2}. \end{aligned}$$

By utilization of Eq. (8), we get following final results:

16 $$\left\{ \begin{aligned} C_{{f_{x} }} Re_{x} ^{{1/2}} = & \frac{{F_{1} ^{{\prime \prime }} (0)}}{{\sqrt \Gamma }},C_{{f_{y} }} Re_{x} ^{{1/2}} = \frac{{F_{2} ^{\prime } (0)}}{{\sqrt \Gamma }}, \\ Nu_{x} Re_{x} ^{{1/2}} = & - \frac{{\left[ {\Theta ^{\prime}(0)} \right]}}{{\sqrt \Gamma }}. \\ \end{aligned} \right.$$

## Numerical solution

For resolving partial differential equations, the finite-element method is an effective approach. This approach’s underlying concept is dividing the domain into small size, known as finite elements. In modern engineering analysis, this method is such a good numerical method that it can be used to solve integral equations in many different fields, including heat transfer and fluid mechanics^42,43^. The first and second essential steps of this method are to assume the piecewise continuous function in order to obtain the solution and to locate the parameters that correspond to the functions in such a way as to minimize the error in the solution^44,45^. With the boundary conditions (12) for the solution of a system of simultaneous partial differential equations as shown in (9–11), we first assume that:

17 $$\begin{aligned} F_1'= H, \end{aligned}$$

Equations (9)–(12) reduces to:

18 $$\begin{aligned} \frac{1}{\chi _1\chi _2}H''+0.5\eta {H}'-\Gamma \eta {H}'+\Gamma ({F_1}{H}'-{H}^2+2\lambda {F_2}-\frac{M^2}{\chi _2}H) = \Gamma (1-\Gamma )\frac{\partial {H}}{\partial \Gamma }, \end{aligned}$$

19 $$\begin{aligned} \frac{1}{\chi _1\chi _2}F_2''+\frac{1}{2}(1-\Gamma )\eta {F_2}'+\Gamma ({F_1}{F_2}'-{H}{F_2}-2\lambda {H}-\frac{M^2}{\chi _2}F_2) = \Gamma (1-\Gamma )\frac{\partial {F_2}}{\partial \Gamma }, \end{aligned}$$

20 $$\begin{aligned} \frac{\chi _3}{\chi _4}\Theta ''+ 0.5\eta (1-\Gamma )P_r\Theta '+P_r\Gamma {F_1}\Theta ' = P_r\Gamma (1-\Gamma )\frac{\partial {\Theta }}{\partial \Gamma }, \end{aligned}$$

21 $$\begin{aligned} \left. \begin{array}{ll} F_1(\Gamma ,\eta =0)=0,\ H(\Gamma ,\eta =0)=1,\ F_2(\Gamma ,\eta =0)=0,\ \Theta (\Gamma ,\eta =0) = 1,\ \Gamma \ge = 0,\ at\ \eta =0,\\ H(\Gamma ,\eta \rightarrow \infty )\rightarrow 0,\ F_2(\Gamma ,\eta \rightarrow \infty )\rightarrow 0,\ \Theta (\Gamma ,\eta \rightarrow \infty )\rightarrow 0,\ \Gamma \ge 0,\ as\ \eta \rightarrow \infty . \end{array}\right\} \end{aligned}$$

**Figure 2:**
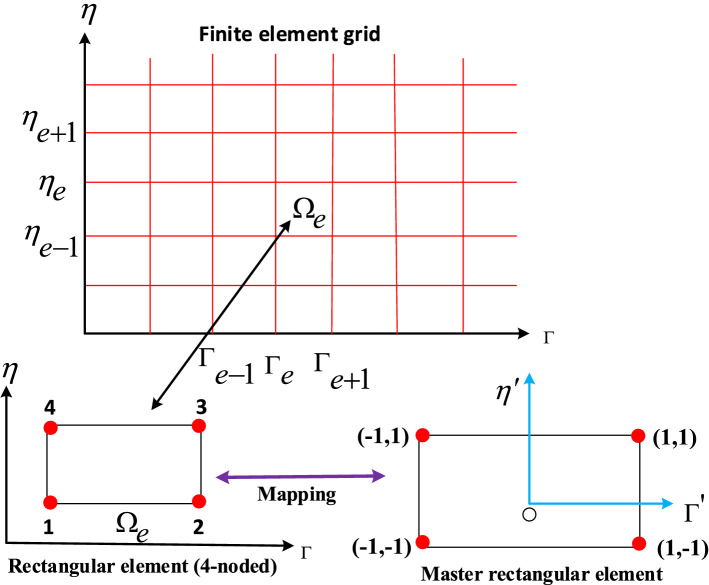
Finite element grid and mesh.

Equations (17)–(20) are given below:

22 $$\begin{aligned}{} & {} \int \limits _{\Omega _e}w_{f_1}\{F_1'-H\}d\Omega _e = 0, \end{aligned}$$

23 $$\begin{aligned}{} & {} \int \limits _{\Omega _e}w_{f_2}\bigg \{\frac{1}{\chi _1\chi _2}H''+\frac{1}{2}(1-\Gamma )\eta {H}'+\Gamma ({F_1}{H}'-{H}^2+2{H}\lambda -\frac{M^2}{\chi _2}H)- \Gamma (1-\Gamma )\frac{\partial {H}}{\partial \Gamma }\bigg \}d\Omega _e= 0, \end{aligned}$$

24 $$\begin{aligned}{} & {} \int \limits _{\Omega _e}w_{f_3}\bigg \{\frac{1}{\chi _1\chi _2}F_2''+\frac{1}{2}(1-\Gamma )\eta {F_2}'+\Gamma ({F_1}{F_2}'-{H}{F_2}-2\lambda {H}-\frac{M^2}{\chi _2}F_2)- \Gamma (1-\Gamma )\frac{\partial {F_2}}{\partial \Gamma }\bigg \}d\Omega _e = 0, \end{aligned}$$

25 $$\begin{aligned}{} & {} \int \limits _{\Omega _e}w_{f_4}\bigg \{\frac{\chi _3}{\chi _4}\Theta ''+ \frac{P_r}{2}(1-\Gamma )\eta \Theta '+P_r\Gamma {F_1}\Theta '- P_r\Gamma (1-\gamma )\frac{\partial \Theta }{\partial \Gamma }\bigg \}d\Omega _e = 0, \end{aligned}$$

where $w_{f_1}$, $w_{f_2}$, $w_{f_3}$, and $w_{f_4}$ are the arbitrary test functions. The grid point of computation domain ($\Omega _e$) is shown in Fig. 2. Equations can be transformed into the finite element model by substituting approximations of the form:

26 $$\begin{aligned} F_1 = \sum _{j=1}^4 F_{1j} \Upsilon _j(\gamma ,\eta ),\ H = \sum _{j=1}^4 H_j \Upsilon _j(\Gamma ,\eta ),\ F_2 = \sum _{j=1}^4 F_{2j} \Upsilon _j(\Gamma ,\eta ),\ \Theta = \sum _{j=1}^4 \Theta _j \Upsilon _j(\Gamma ,\eta ). \end{aligned}$$

In above mentioned mathematical relation, $\Upsilon _j$ show functions of linear interpolation. The rectangular elements are given below:

27 $$\begin{aligned} \left. \begin{array}{ll} \Upsilon _1 = \frac{(\Gamma _{e+1}-\Gamma )(\eta _{e+1}-\eta )}{(\Gamma _{e+1}-\Gamma _e)(\eta _{e+1}-\eta _e)},\ \Upsilon _2 = \frac{(\Gamma -\Gamma _e)(\eta _{e+1}-\eta )}{(\Gamma _{e+1}-\Gamma _e)(\eta _{e+1}-\eta _e)},\\ \Upsilon _3 = \frac{(\Gamma -\Gamma _e)(\eta -\eta _e)}{(\Gamma _{e+1}-\Gamma _e)(\eta _{e+1}-\eta _e)},\ \Upsilon _4 = \frac{(\Gamma _{e+1}-\Gamma )(\eta -\eta _e)}{(\Gamma _{e+1}-\Gamma _e)(\eta _{e+1}-\eta _e)}. \end{array}\right\} \end{aligned}$$

The final equations model for finite element is shown below:

28 $$\begin{aligned} \begin{bmatrix} [L^{11}] &{} [L^{12}] &{} [L^{13}] &{} [L^{14}] \\ [L^{21}] &{} [L^{22}] &{} [L^{23}] &{} [L^{24}] \\ [L^{31}] &{} [L^{32}] &{} [L^{33}] &{}[ L^{34}] \\ [L^{41}] &{} [L^{42}] &{} [L^{43}] &{} [L^{44}] \end{bmatrix} \begin{bmatrix} \{F_1\}\\ \{H\}\\ \{F_2\} \\ \{\Theta \} \end{bmatrix} = \begin{bmatrix} \{R_1\} \\ \{R_2\}\\ \{R_3\} \\ \{R_4\} \end{bmatrix} \end{aligned}$$

where $[L_{mn}]$ and $[R_m]$ (m, n = 1, 2, 3, 4) are defined as:

$$\begin{aligned} L^{11}_{ij}= & {} \int \limits _{\Omega _e} \Upsilon _i\frac{d\Upsilon _j}{d\eta }d\Omega _e, L^{12}_{ij} = -\int \limits _{\Omega _e} \Upsilon _i\Upsilon _jd\Omega _e, L^{13}_{ij} = L^{14}_{ij} = L^{15}_{ij} = L^{21}_{ij} = L^{24}_{ij} = 0, \\ L^{22}_{ij}= & {} -\frac{1}{\chi _1\chi _2}\int \limits _{\Omega _e} \frac{d\Upsilon _i}{d\eta } \frac{d\Upsilon _j}{d\eta }d\Omega _e +\frac{1}{2}(1-\Gamma )\eta \int \limits _{\Omega _e}\Upsilon _i \frac{d\Upsilon _j}{d\eta }d\Omega _e+\Gamma \int \limits _{\Omega _e} \bar{F_1}\Upsilon _i\frac{d\Upsilon _j}{d\eta }d\Omega _e-\Gamma \int \limits _{\Omega _e}\bar{H}\Upsilon _i \Upsilon _j d\Omega _e- \frac{M^2}{\chi _2}\Gamma \int \limits _{\Omega _e}\Upsilon _i \Upsilon _j d\Omega _e,\\{} & {} -\Gamma (1-\Gamma )\int \limits _{\Omega _e}\Upsilon _i \frac{d\Upsilon _j}{d\Gamma }d\Omega _e,\ L^{23}_{ij} = 2\lambda \Gamma \int \limits _{\Omega _e}\Upsilon _i \Upsilon _j d\Omega _e,\ L^{31}_{ij}=L^{34}_{ij}=0,\ L^{32}_{ij}=-2\lambda \Gamma \int \limits _{\Omega _e}\Upsilon _i \Upsilon _jd\Omega _e,\\ L^{33}_{ij}= & {} -\frac{1}{\chi _1\chi _2}\int \limits _{\Omega _e}\frac{d\Upsilon _i}{d\eta }\frac{d\Upsilon _j}{d\eta }d\Omega _e +\frac{1}{2}(1-\Gamma )\eta \int \limits _{\Omega _e}\Upsilon _i \frac{d\Upsilon _j}{d\eta }d\Omega _e+\Gamma \int \limits _{\Omega _e} \bar{F_1}\Upsilon _i\frac{d\Upsilon _j}{d\eta }d\Omega _e -\Gamma \int \limits _{\Omega _e}\bar{H}\Upsilon _i \Upsilon _j d\Omega _e\\{} & {} -\Gamma (1-\Gamma )\int \limits _{\Omega _e}\Upsilon _i \frac{d\Upsilon _j}{d\Gamma }d\Omega _e- \frac{M^2}{\chi _2}\Gamma \int \limits _{\Omega _e}\Upsilon _i \Upsilon _j d\Omega _e, L^{41}_{ij} = L^{42}_{ij} = L^{43}_{ij} = 0,\\ L^{44}_{ij}= & {} -\frac{\chi _3}{\chi _4}\int \limits _{\Omega _e} \frac{d\Upsilon _i}{d\eta } \frac{d\Upsilon _j}{d\eta }d\Omega _e + \frac{Pr}{2}(1-\Gamma )\eta \int \limits _{\Omega _e}\Upsilon _i \frac{d\Upsilon _j}{d\eta }d\Omega _e +P_r\zeta \int \limits _{\Omega _e} \bar{F_1}\Upsilon _i\frac{d\Upsilon _j}{d\eta }d\Omega _e -P_r\Gamma (1-\Gamma )\int \limits _{\Omega _e}\Upsilon _i \frac{d\Upsilon _j}{d\Gamma }d\Omega _e, \end{aligned}$$

and

29 $$\begin{aligned} R^1_i = 0, \ R^2_i = -\oint \limits _{\Gamma _e} \Upsilon _i n_{\eta }\frac{\partial {H}}{\partial \eta } \,ds, \ R^3_i = -\oint \limits _{\Gamma _e} \Upsilon _i n_{\eta }\frac{\partial {F_2}}{\partial \eta } \,ds, \ R^4_i = -\oint \limits _{\Gamma _e} \Upsilon _i n_{\eta }\frac{\partial \Theta }{\partial \eta }\,ds. \end{aligned}$$

where $\bar{F}_1 = \sum _{j=1}^4 \bar{F}_{1j} \Upsilon _j$, $\bar{H} = \sum _{j=1}^4 \bar{H}_j \Upsilon _j$, $\bar{F}_2 = \sum _{j=1}^4 \bar{F}_{2j}\Upsilon _j$, and $\bar{\Theta }' = \sum _{j=1}^4 \bar{\Theta }'_j\Upsilon _j$ assumed to be the known. The 4 functions are compute at each node. The final system of equations are non-linear forms after assembly, so linearize this system via an iterative algorithm subject to the required precision of $10^{-5}$.

## Results and discussion

**Table 3:** Study of different size of grid independent when $\Gamma = 1.0$.

Grid size	$-F_1''(\zeta ,0)$	$-F_2'(\zeta ,0)$	$-\Theta '(\zeta ,0)$
10 $\times$ 10	2.2996	1.1554	1.3276
30 $\times$ 30	2.2172	1.2295	1.2298
50 $\times$ 50	2.2130	1.2168	1.2237
80 $\times$ 80	2.2122	2.2122	1.2211
100 $\times$ 100	2.2120	1.2103	1.2206
130 $\times$ 130	2.2119	1.2100	1.2204

**Table 4:** Comparative of $-F_{1}^{'}(0)$ and $-F_{2}^{''}(0)$ against various strength of $\lambda$ at $\Gamma =1.0$ subject to no effect of others parameters.

$\lambda$	Ali et al.^40^		Wang^46^		Present	
	$-F_{1}^{''}(0)$	$-F_{2}^{'}(0)$	$-F_{1}^{''}(0)$	$-F_{2}^{'}(0)$	$-F_{1}^{''}(0)$	$-F_{2}^{'}(0)$
0.0	1.00000	0.00000	1.0000	0.0000	1.00000	0.00000
1.0	1.32501	0.83715	1.3250	0.8371	1.32501	0.83715
2.0	1.65232	1.28732	1.6523	1.2873	1.65232	1.28732
5.0	2.39026	2.15024	–	–	2.39026	2.15024

**Table 5:** Comparison of $-\theta '(0)$ against various strength of $\lambda$ at $\Gamma =1.0$ subject to no effect of others parameters.

$\lambda$	Adnan et al.^48^	Ali et al.^47^	Current outcomes	
	$M = 0.0, Pr = 2.0$	$M = Pr = 2.0$	$M = 0.0, Pr = 2.0$	$M = Pr = 2.0$
0.0	0.911	0.6682	0.911068	0.668210
0.5	0.853	0.6627	0.853430	0.662675
1.0	0.770	0.6483	0.770279	0.648282
2.0	0.638	0.6030	0.638053	0.603031

Using a graphical representation, the physical effects of the various flow parameters, including the rotating, magnetic, and time dependent parameters, on the dimensionless fluid flow velocity and the temperature are shown. The effects of particle aggregation suspensions with microscopic particles and a fluid that is susceptible to Lorentz and Coriolis forces have been demonstrated by our findings. The each graph show two types of curves for nanoparticles aggregation ($\Phi _{int} \ne 1.0$) and non aggregation ($\Phi _{int} = 1.0$). Throughout the computation, the others involved parameters default values are: $P_r = 6.2$, $M = 1.0$, $\lambda =1.0$, $D = 1.8$, $\Phi = 0.01$, $\Phi _{max} = 0.650$, and $R_a/R_p = 3.34$. A grid independence study is carried out in order to examine the Galerkin finite element approach’s reliability and validity. We finalized all of the results on a grid that is $100\times 100$ because the problem input is divided into different mesh densities and there is no more variation after that (see Table 3). In some cases, a validation with previous studies is presented in Tables 3 and 4 to demonstrate that the current results are valid and reliable. As shown, the current results are very similar to the published ones. Table 4 displays the friction factors as well as the primary and secondary directions ($-F_1''(0)$ & $-F_2(0))$ in response to increasing inputs of ($\lambda = 0, 1, 2, 5)$ at ($\Gamma = 1.0)$. The findings are very consistent with those analyzed by Wang et al.^46^ and Ali et al.^40^. In addition, the $-\Theta '(0)$ finding are compared between Bagh et al.^47^ and Adnan et al.^48^, who present finite element results against rising inputs of $M\ \& \ \lambda$, and discovered that they are in an excellent agreement in Table 5. Therefore, numerical calculations can be validated, and the Matlab created Finite Element Computations have a high rate of convergence.

**Figure 3:**
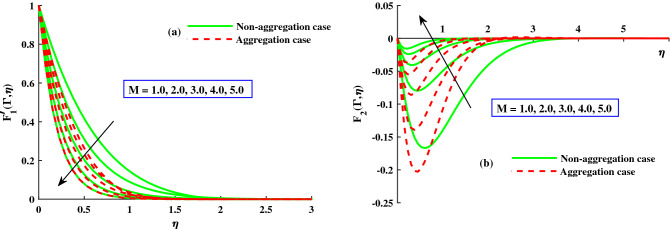
The influence of *M* on $F_{1}^{'}(\Gamma ,\eta )$ in axial, and $F_{2}^{'}(\Gamma ,\eta )$ in transverse at $\Gamma =1$.

**Figure 4:**
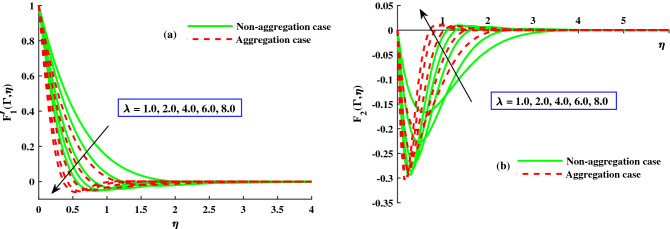
The influence of $\lambda$ on $F_{1}^{'}(\Gamma ,\eta ) \& \ F_{2}^{'}(\Gamma ,\eta )$ at $\Gamma = 1$.

**Figure 5:**
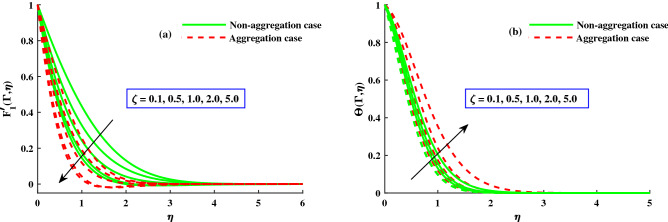
The influence of $\zeta$ on $F_{1}^{'}(\Gamma ,\eta ) \& \ \Theta (\Gamma ,\eta )$ at $\Gamma =1$.

The response of the variables in the boundary layer to the magnetic field (*M*), is depicted in Fig. 3a,b. An increase in *M* results in a significant decrease in the primary velocity $F_1'(\Gamma ,\eta )$). With increasing magnetic field, the $F_2'(\Gamma ,\eta )$ also experiences a significant magnitude decrease. The fluid flow in xy plane are both significantly slowed down by the Lorentzian drag forces $- \frac{M^2}{\chi _2}F_1'$ and $-\frac{M^2}{\chi _2}F_2$ in Eqs. (9) and (10). These magnetic forces are produced in the plane of the sheet, which are transverse to axial direction. The primary velocity never reversal flow because the surface is extend in the x-direction. However, as evidenced by the negative values in Fig. 3b, the secondary fluid flow which is perpendicular over the primary fluid flow, significant experiences backflow. The $F_1'(\Gamma , \eta )$ profiles indicate that when the magnetic field is at its weakest, $M = 1.0$, the Lorentz resistance is approximately the same as the viscous hydrodynamic force in the nanofluid. The primary and second velocity components are all affected by the rotational parameter $\lambda$, as shown in Fig. 4a,b. A growing in the rotational parameter is accompanied by a significant decrease in the primary flow velocity. The magnitude of secondary velocity also decreases when the rotation parameter is increased. As previously stated, the crossflow effects cause the secondary flow to be negative, or backflow. In this case, in the x-direction momentum development is aided by the sheet’s stretching direction, while in the y-direction momentum development is counteracts. In Eqs. (9) and (10) respectively, the Coriolis forces are $+2\lambda {F_2}$ and $-2\lambda {F_1'}$. Even though the body force is positive in first momentum equation, the $F_2'(\Gamma ,\eta )$ is negative, so the primary momentum field is affected negatively overall. As a result, as the rotational parameter escalate, the primary velocity recedes (Fig. 4a). The flow variables’ responses to the unsteadiness parameter, $\zeta$, are depicted in Fig. 5a,b. The predominant pattern is that primary velocity decreases as unsteadiness increases (Fig. 4a). Since the unsteadiness effect has been dampened out, the ($F_1'(\Gamma , \eta )$) is effectively stabilized by a decline in the time dependent parameter. On the other hand, we notice that the regime’s temperatures rise significantly with increasing unsteadiness parameter (Fig. 5b). According to Ali et al.^38^, for transient Newtonian convection surface extending flow, this is due to a upgrade in unsteady convection currents, which enhances boundary layer thermal diffusion. In addition, the model and nanoparticle aggregation have a lesser distribution of ($F_1'(\Gamma , \eta )$) and ($F_2'(\Gamma , \eta )$), whereas the distribution of $F_1'(\Gamma , \eta )\ \& \ F_2'(\Gamma , \eta )$ is slightly higher than that of non-aggregated nanoparticles case. Physically, the effective viscosity^49^ increased as a result of the aggregation of nanoparticles, and the increasing strength of the viscosity slowed the fluid velocity^50^.

**Figure 6:**
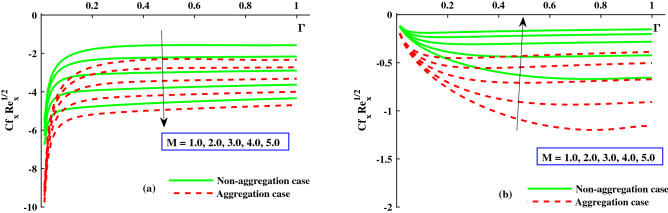
The influence of $\lambda$ on $Cf_xRe^{1/2}_x$ in x-direction, and $Cf_yRe^{1/2}_y$ in y-direction.

**Figure 7:**
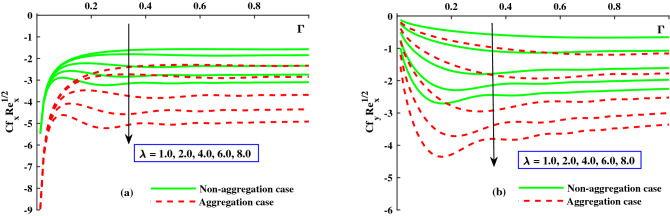
The influence of *M* on $Cf_xRe^{1/2}_x$ in x-direction, and $Cf_yRe^{1/2}_y$ in y-direction.

**Figure 8:**
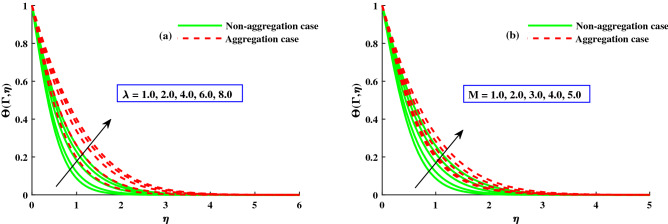
The influence of *M* & $\lambda$ on $\Theta (\Gamma ,\eta )$ at $\Gamma =1$.

**Figure 9:**
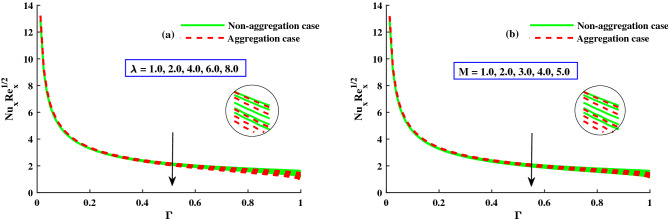
The influence of $Nu_xRe_x^1/2$ via *M* & $\lambda$.

Figure 6a,b depicts the distribution of friction factors $(C_{f_x}{Re_x}^{1/2})$ and $(C_{f_y}{Re_x}^{1/2})$ against upgrading values of the parameters $\Gamma$(0:0.3:1.0) and *M*(1.0:1.0:5.0). Figure 6a demonstrates that for increasing $\Gamma (0)rightarrow 1$, the $(C_{f_x}{Re_x}^{1/2})$ increases steadily to a fixed rate and does not change noticeably; however, for strengthen *M*, a significant decrease in the axial friction is observed. Figure 6b shows that the $(C_{f_y}{Re_x}^{1/2})$ decreases steadily as $\Gamma (0\rightarrow 1$ increases until it reaches a constant rate, at which point there is no discernible difference. However, when *M* is improved, the significance difference near the boundary surface is noted. Figure 7a,b shows that for increasing $\Gamma (0\rightarrow 1$, the $(C_{f_x}{Re_x}^{1/2})$ is gradually raised until it approach a constant rate, after which no significant change is observed. On the other hand, for increasing $\lambda$, both the $(C_{f_x}{Re_x}^{1/2})$ and the $(C_{f_y}{Re_x}^{1/2})$ are significantly decreased. In addition, the ranges of $(C_{f_x}{Re_x}^{1/2})$ and $(C_{f_y}{Re_x}^{1/2})$ for the model with nanoparticle aggregation are shown to have a distribution that is significantly lower than that of the case with non-aggregated nanoparticles. Figures 8 and 9 depict the distribution of $\Theta (\Gamma ,\eta )$ for various parameters. As can be seen in Fig. 8a, the temperature distribution, $\Theta (\Gamma , \eta )$, was enhanced by the magnetic parameter. The temperature profile depicted in Fig. 8a is controlled by a net force known as the Lorentz force subject to internal electric force, and the external magnetic field, whereas the thermal boundary layer thickness recedes while the $\lambda$ value increases, as shown in Fig. 8b. Figure 9a,b depicts the sketches of the local Nusselt number $(Nu_{x}{Re_x}^{1/2})$ for *M*(1:1:5) and $\lambda (1:2:8)$. The distribution of $(Nu_{x}{Re_x}^{1/2})$ gradually decreases as *M* and $\lambda$ grow. The $(Nu_{x}{Re_x}^{1/2})$ decreases significantly in the nanoparticle aggregation model, while the distribution of $(Nu_{x}{Re_x}^{1/2})$ is slightly higher than in the case of nanoparticles non-aggregation.

## Conclusions

A theoretical model for predicting the thermal conductivity of nanofluids has been developed by taking into account the structure of the aggregates and nanoparticles, as well as the physical properties of the base liquid and the nanoparticles. Effective nanofluid thermal conductivity and viscosity for homogeneous models and nanoparticle aggregation were investigated by the authors. It is reasonable to draw the following main findings based on present report analysis:

1. The nanoparticles aggregation causes a decline in magnitude of both primary and secondary velocities, and lesser the strength of $C_{f_x}{Re_x}^{1/2}$ and $C_{f_y}{Re_x}^{1/2}$.
2. The fact that the aggregation model has a temperature profile that is higher than that of typical model and a significant decline in $Nu_{x}{Re_x}^{1/2}$.
3. The magnitudes of axial momentum and transverse momentum decrease when the Coriolis and Lorentz strengths are exceeded, and
  improve thermal performance of base fluid and a negative influence on $Nu_{x}{Re_x}^{1/2}$.
  Increase the value of the skin friction factor, $Cf_xRe_x^{1/2}$.

## Data Availability

The data used to support the findings of this study are available from the corresponding author upon request.
