# Author Correction: Influence of nanoparticles aggregation and Lorentz force on the dynamics of water-titanium dioxide nanoparticles on a rotating surface using finite element simulation

**DOI:** 10.1038/s41598-023-33068-4

**Published:** 2023-04-12

**Authors:** Bagh Ali, Imran Siddique, Hijaz Ahmad, Sameh Askar

**Affiliations:** 1 School of Mechanical Engineering and Automation, Harbin Institute of Technology, Shenzhen, 518055 China; 2 Faculty of Computer Science and Information Technology, Superior University, Lahore, 54000 Pakistan; 3 Department of Mathematics, University of Management and Technology, Lahore, 54770 Pakistan; 4 Section of Mathematics, International Telematic University Uninettuno, Corso Vittorio Emanuele II, 39, 00186 Rome, Italy; 5 Department of Statistics and Operations Research, College of Science, King Saud University, P.O. Box 2455, Riyadh, 11451 Saudi Arabia; 6 Near East University, Operational Research Center in Healthcare, Near East Boulevard, 10, 99138 Nicosia/Mersin, Turkey

Correction to: *Scientific Reports* 10.1038/s41598-023-31771-w, published online 22 March 2023

The original version of this Article omitted an affiliation for Hijaz Ahmad. The correct affiliations are listed below.

Section of Mathematics, International Telematic University Uninettuno, Corso Vittorio Emanuele II, 39, 00186 Roma, Italy

Near East University, Operational Research Center in Healthcare, Near East Boulevard, PC: 99138 Nicosia/Mersin 10, Turkey

The original Article has been corrected.

